# Wearable smartwatches for atrial fibrillation detection and burden estimation after ablation: comparison with continuous monitoring

**DOI:** 10.1093/europace/euaf280

**Published:** 2025-11-10

**Authors:** Martin Aguilar, Laurent Macle, Ralph Chamieh, Paul Khairy, Marc W Deyell, Richard G Bennett, Jason G Andrade

**Affiliations:** Montreal Heart Institute, Department of Medicine, Université de Montréal, 5000 Rue Bélanger, Montréal, QC, Canada H1T 1C8; Montreal Heart Institute, Department of Medicine, Université de Montréal, 5000 Rue Bélanger, Montréal, QC, Canada H1T 1C8; Montreal Heart Institute, Department of Medicine, Université de Montréal, 5000 Rue Bélanger, Montréal, QC, Canada H1T 1C8; Montreal Heart Institute, Department of Medicine, Université de Montréal, 5000 Rue Bélanger, Montréal, QC, Canada H1T 1C8; Department of Medicine, Dilawri Cardiovascular Institute, 2775 Laurel St Vancouver, Canada BC V5Z 1M9; Center for Cardiovascular Innovation, Life Sciences Centre, 2350 Health Sciences Mall, Vancouver, Canada BC V6T 1Z3; Department of Medicine, Dilawri Cardiovascular Institute, 2775 Laurel St Vancouver, Canada BC V5Z 1M9; Center for Cardiovascular Innovation, Life Sciences Centre, 2350 Health Sciences Mall, Vancouver, Canada BC V6T 1Z3; Montreal Heart Institute, Department of Medicine, Université de Montréal, 5000 Rue Bélanger, Montréal, QC, Canada H1T 1C8; Department of Medicine, Dilawri Cardiovascular Institute, 2775 Laurel St Vancouver, Canada BC V5Z 1M9; Center for Cardiovascular Innovation, Life Sciences Centre, 2350 Health Sciences Mall, Vancouver, Canada BC V6T 1Z3

**Keywords:** Atrial fibrillation, Catheter ablation, AF burden, Wearables, Implantable cardiac monitor

## Abstract

**Aims:**

Wearable ECG-enabled smartwatches have been validated for atrial fibrillation (AF) screening, but their accuracy for monitoring AF recurrence and quantifying AF burden after catheter ablation is uncertain. To evaluate the simulated performance of three commercial smartwatch algorithms for AF recurrence detection and AF burden estimation compared with implantable cardiac monitors (ICMs).

**Methods and results:**

Using continuous ICM data from 346 patients in the CIRCA-DOSE trial, we simulated three smartwatch algorithms, assuming daytime wear (8:00 AM–10:00 p.m.). We also simulated commonly-used non-invasive intermittent rhythm monitoring strategies. Primary endpoints were sensitivity for arrhythmia recurrence and correlation with ICM-derived AF burden. Analyses were stratified by daily wear time and patient activity. AF recurrence occurred in 47.1% of patients. Simulated detection sensitivities were 82.2% (Apple Watch AF Burden), 70.6% (Apple Watch IRN), and 64.4% (Fitbit IHRD), compared with 15.8–64.6% for simulated intermittent AECG monitors. Wearables outperformed commonly-used Holter/patch monitoring strategies. AF burden correlation with ICM exceeded r = 0.97 for all algorithms. Among missed recurrences, median AF burden was <0.02%. Longer daily wear improved sensitivity (>90% with 24-hour use), whereas patient activity modestly reduced detection.

**Conclusion:**

Smartwatch-based AF detection algorithms demonstrate strong correlation with ICM-derived AF burden and clinically good sensitivity for recurrence detection, outperforming conventional non-invasive strategies. These findings support the integration of wearables as a scalable alternative for post-ablation rhythm monitoring.

## Graphical Abstract

**Graphical Abstract:**
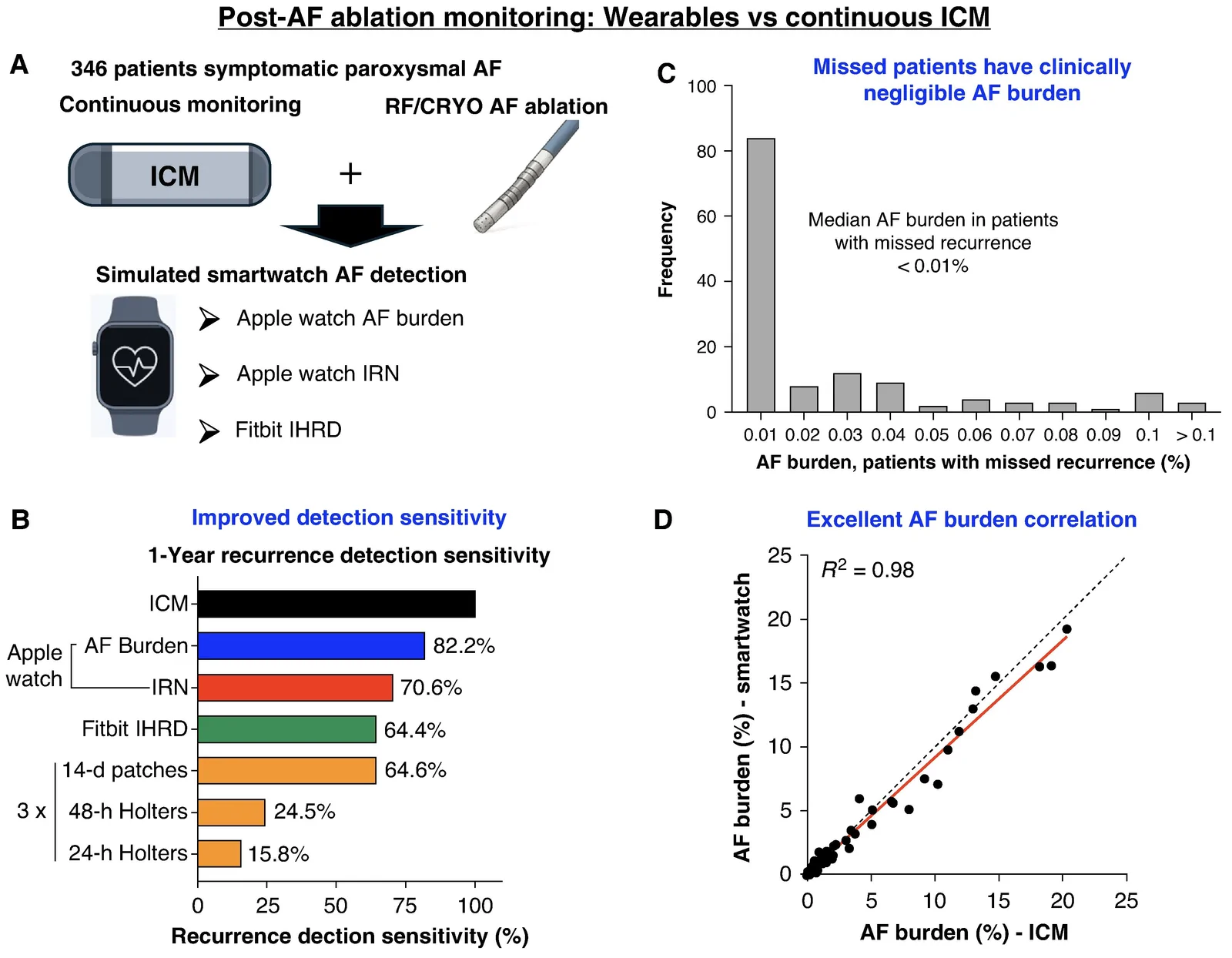
(A) Study design: 346 patients with paroxysmal AF in the CIRCA-DOSE trial underwent continuous monitoring with an implantable cardiac monitor (ICM) after AF ablation. We simulated smartwatch detection (Apple Watch AF Burden, Apple Watch Irregular Rhythm Notification [IRN], Fitbit Irregular Heart Rhythm Detection [IHRD]). (B) Detection sensitivity for 1-year AF recurrence: wearable algorithms substantially outperform conventional intermittent monitoring (14-day patch, 48-hour and 24-hour Holter). (C) Missed recurrences with wearables had extremely low AF burden (median <0.02%), well below clinically significant thresholds. (D) The correlation between AF burden measured by wearables and ICM was excellent (Pearson r > 0.97), confirming that smartwatch-based burden estimation is accurate and robust.

What’s new?

- This is the first study to benchmark commercially available smartwatch atrial fibrillation (AF) detection algorithms directly against continuous implantable cardiac monitoring in the post-ablation setting.
- Smartwatch algorithms (Apple Watch AF Burden, Apple Watch IRN, Fitbit IHRD) demonstrated good sensitivity for AF recurrence detection (64–82%), outperforming conventional Holter and patch monitoring strategies.
- Wearable-estimated AF burden was highly correlated with implantable cardiac monitor–derived burden (Pearson r >0.97), even under conditions of variable daily use and patient activity.
- Missed arrhythmia recurrences with wearables were of extremely low burden (median <0.02%), suggesting limited clinical relevance.
- These results support the integration of consumer smartwatches as a scalable, non-invasive alternative for post-ablation rhythm surveillance and AF burden assessment.

## Introduction

Atrial fibrillation (AF) is the most prevalent sustained arrhythmia seen in clinical practice and is associated with increased morbidity, mortality and healthcare utilization.^1^ Catheter ablation is an established therapy for symptomatic AF; however, effective post-procedural rhythm monitoring is critical to assess arrhythmia recurrence and quantify AF burden. Current guidelines acknowledge the limitations of traditional follow-up methods, yet the optimal monitoring strategy after ablation has not been clearly defined.^2^ Importantly, AF detection and AF burden assessment are highly dependent on the diagnostic modality applied,^3^ as emphasized in the recent EHRA position paper on AF screening strategies.^4^ Increasingly sophisticated approaches, including digital devices, are being developed to extend monitoring duration and improve diagnostic yield.

Intermittent, short-duration ambulatory ECG monitoring—such as Holter or event monitors—is widely used but suffers from low sensitivity, often underdetecting asymptomatic or low-burden episodes and consequently overestimating procedural success.^3,5^ While implantable cardiac monitors (ICMs) provide continuous, high-fidelity rhythm surveillance and are considered the gold standard, their invasive nature, cost, and limited scalability restrict their routine clinical use.

In recent years, smartwatches equipped with photoplethysmography (PPG) or electrocardiography (ECG) have emerged as promising alternatives for non-invasive, direct-to-consumer rhythm monitoring.^6^ With growing consumer usage, one in three U.S. adults reported using a wearable device—wearables represent an attractive, non-invasive, and scalable method of rhythm monitoring.^7^ However, their clinical utility for long-term arrhythmia monitoring and quantification of AF burden, particularly in the context of catheter ablation, is poorly defined.

In this study, we used continuous rhythm data from the CIRCA-DOSE randomized trial to simulate how three commercial wearable algorithms would identify arrhythmia recurrence and estimate AF burden. Using ICM as the gold standard, we assessed the detection sensitivity and accuracy of AF burden in these wearable-based algorithms for post-AF ablation follow-up care. This study aimed to provide the first systematic evaluation of commercially available smartwatch-based AF detection algorithms against continuous monitoring in a post-AF ablation population, and to compare their performance with that of commonly used non-invasive intermittent monitoring strategies.

## Methods

### Study population

We analysed 346 patients undergoing a first catheter ablation for symptomatic paroxysmal AF in the Cryoballoon or Irrigated Radiofrequency Catheter Ablation randomized controlled trial (CIRCA-DOSE NCT01913522).^8,9^ All patients underwent insertion of an ICM (Reveal LINQ, Medtronic, Minneapolis) before ablation. The institutional ethics committee approved the study, along with the review board of each participating centre. The data supporting this study's findings are available from the corresponding author upon reasonable request.

### Simulated AF detection by wearables

The ICM-derived AF burden was calculated as the per cent time in AF from the end of the blanking period (day 90) to the end of the study (day 365). We then simulated the AF detection delivered by (i) the Apple Watch AF burden algorithm, (ii) the Apple Watch Irregular Rhythm Notification algorithm and (iii) the Fitbit Irregular Heart Rhythm Detection algorithm. The Apple Watch AF burden algorithm was simulated as follows: A 1-minute window was checked every 15 min. The AF burden was calculated as the number of checks with AF divided by the total number of checks.^10^ The Apple Watch Irregular Rhythm Notification (IRN) algorithm was simulated as follows: a 1-minute window was checked every 2 h. If AF was present, a 1-minute window was checked every 15 min. If 3 out of 5 of these checks had AF, an Irregular Rhythm Notification alert was generated. The AF burden was calculated as the number of alerts divided by the total number of q2-hour checks.^10^ The Fitbit Irregular Heart Rhythm Detection (IHRD) algorithm was simulated as follows: a 5-minute window was checked every 2.5 min. If AF was detected on 11 consecutive checks, an Irregular Heart Rhythm Detection alert was generated. The AF burden was calculated as the total number of alerts divided by the number of q-2.5 min checks.^11^ The simulations had patients wear the watch from 8:00 a.m. to 10:00 p.m., unless otherwise stated.

### Statistical analyses

Continuous variables are presented as medians with interquartile ranges (IQR) and were compared using the Wilcoxon signed-rank test for paired data. Categorical variables are expressed as frequencies and percentages. Kaplan-Meier estimates were used to calculate 1-year arrhythmia-free survival rates, and comparisons between monitoring strategies were made using the log-rank test. The detection sensitivity of wearable algorithms for arrhythmia recurrence was calculated relative to the ICM reference standard.

To assess the agreement between AF burden estimates derived from each wearable algorithm and the ICM, we used Pearson correlation coefficients. Analyses were repeated stratified by daily watch usage (1 to 15 h per day) and by simulated patient activity levels (0%, 25%, 50%, and 75% probability of missing rhythm checks). Differences in AF burden distributions between methods were assessed using paired nonparametric tests. A two-tailed *P*-value <0.05 was considered statistically significant. All statistical analyses were performed using Prism 10 (GraphPad Software; San Diego, CA) and Matlab R2023a (MathWorks; Natick, MA). The data underlying this article will be shared on reasonable request to the corresponding author.

## Results

### Arrhythmia recurrence detection and AF burden

**Figure 1:**
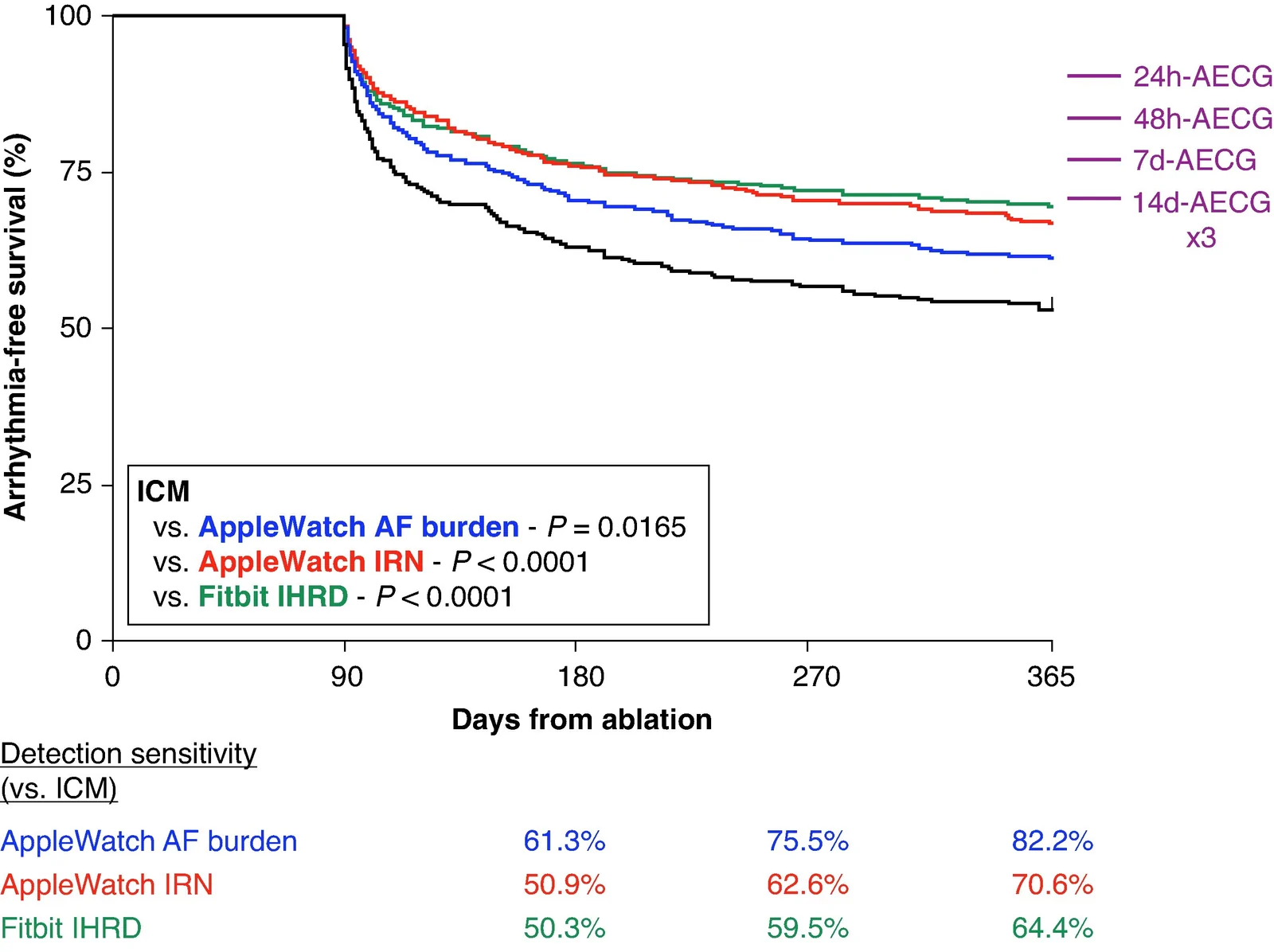
Kaplan–Meier analysis of arrhythmia-free survival. One-year arrhythmia-free survival after atrial fibrillation (AF) ablation as detected by implantable cardiac monitor (ICM; black) and by simulated wearable algorithms: Apple Watch AF Burden (blue), Apple Watch Irregular Rhythm Notification (IRN; red), and Fitbit Irregular Heart Rhythm Detection (IHRD; green). Wearables detect significantly more recurrences than standard non-invasive monitors, narrowing the gap with continuous ICM surveillance. The purple lines indicate survival estimates from simulated non-invasive ambulatory ECG monitoring strategies (24-hour, 48-hour, 7-day, and 14-day AECG) at 3, 6, and 12 months. P values represent log-rank comparisons with ICM.

A total of 163 patients (47.1%) experienced a first recurrence of any (symptomatic or asymptomatic) atrial tachyarrhythmia (atrial fibrillation, atrial flutter, or atrial tachycardia) between post-ablation day 90 and 365. The arrhythmia-free survival at 1 year using ICM was 52.6% (95% CI, 47.5–58.0%). The arrhythmia-free survival at 1 year using the Apple Watch AF burden algorithm, the Apple Watch IRN algorithm, and the Fitbit IHRD algorithm were 61.3% (95% CI, 55.9–66.2%, *P* = 0.0165 vs. ICM), 66.8% (95% CI, 61.5–71.5%, *P* < 0.0001 vs. ICM) and 69.7% (95% CI, 64.5–74.2%, *P* < 0.0001), respectively (*Figure 1*). This translated to detection sensitivities (vs. ICM) of 82.2%, 70.6% and 64.4%, respectively. As a comparator, the 1-year arrhythmia-free survival obtained in the same patient population by simulating three 24-hour, 48-hour and, 7-day and 14-day ambulatory ECG (AECG) monitors at 3, 6 and 12 months was 92.5% (95% CI, 90.2–95.8), 88.4% (95% CI, 85.5–92.3%), 79.8% (95% CI, 75.9–84.4%) and 69.4% (64.8–74.6%), respectively. The sensitivities for the AECG monitors were 15.8% (95% CI, 8.9–20.7%), 24.5% (95% CI, 16.2–30.6%), 41.6% (95% CI, 32.9–50.8%) and 64.6% (95% CI, 53.6–74.3%), respectively.^5^

**Figure 2:**
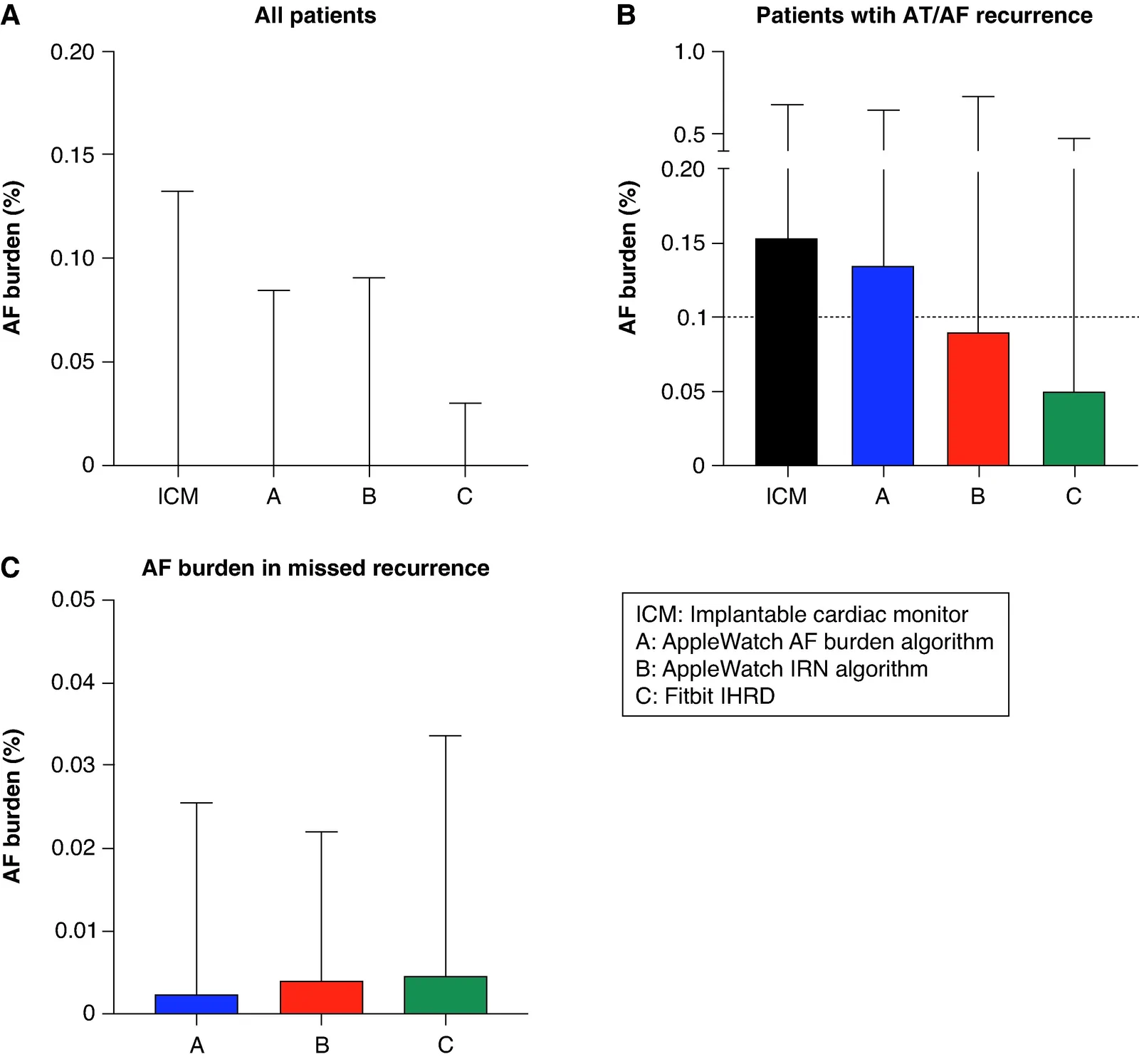
Comparison of AF burden estimates across monitoring strategies. (*A*) AF burden for the entire study population calculated using the implantable cardiac monitor (ICM; Black), the Apple Watch AF burden algorithm (Blue), the Apple Watch IRN algorithm (Red) and the Fitbit IHRD algorithm (Green). (*B*) AF burden for those patients with AT/AF recurrence (non-zero AF burden) calculated with the ICM, and for the Apple Watch AF burden algorithm, the Apple Watch IRN algorithm and the Fitbit IHRD algorithm. (*C*) ICM-derived AF burden in those patients with missed recurrence detection with the Apple Watch AF burden algorithm, the Apple Watch IRN algorithm and the Fitbit IHRD algorithm.

The median ICM-derived AF burden was 0.00% (IQR, 0.00–0.13%). The median AF burden calculated using the Apple Watch AF burden algorithm, the Apple Watch IRN algorithm and the Fitbit IHRD algorithm was 0.00% (IQR, 0.00–0.08%), 0.00% (IQR, 0.00–0.09%), and 0.00% (IQR, 0.00–0.03%), respectively (*Figure 2A*). Considering only patients with AT/AF recurrence (non-zero AF burden), the median AF burden calculated with ICM was 0.15% (IQR, 0.02–0.68%), the Apple Watch AF burden algorithm was 0.13% (IQR, 0.01–0.64%; *P* = 0.0071 vs. ICM), the Apple Watch IRN algorithm was 0.09% (IQR, 0.00–0.73%; *P* = 0.0010 vs. ICM), and the Fitbit IHRD algorithm was 0.05% (IQR, 0.00–0.48%; *P* < 0.0001 vs. ICM), respectively (*Figure 2B*).

The median ICM-derived AF burden in those patients missed by the Apple Watch AF burden algorithm, the Apple Watch IRN algorithm and the Fitbit IHRD algorithm was 0.003% (IQR, 0.001–0.026%), 0.004% (IQR, 0.001–0.022%) and 0.004% (IQR, 0.002–0.034%), respectively (*Figure 2C*). There were 0/89 (0%), 1/89 (1.1%) and 2/89 (2.2%) patients with ICM-derived AF burden >0.1% that were missed by the Apple Watch AF burden algorithm, the Apple Watch IRN algorithm and the Fitbit IHRD algorithm.

**Figure 3:**
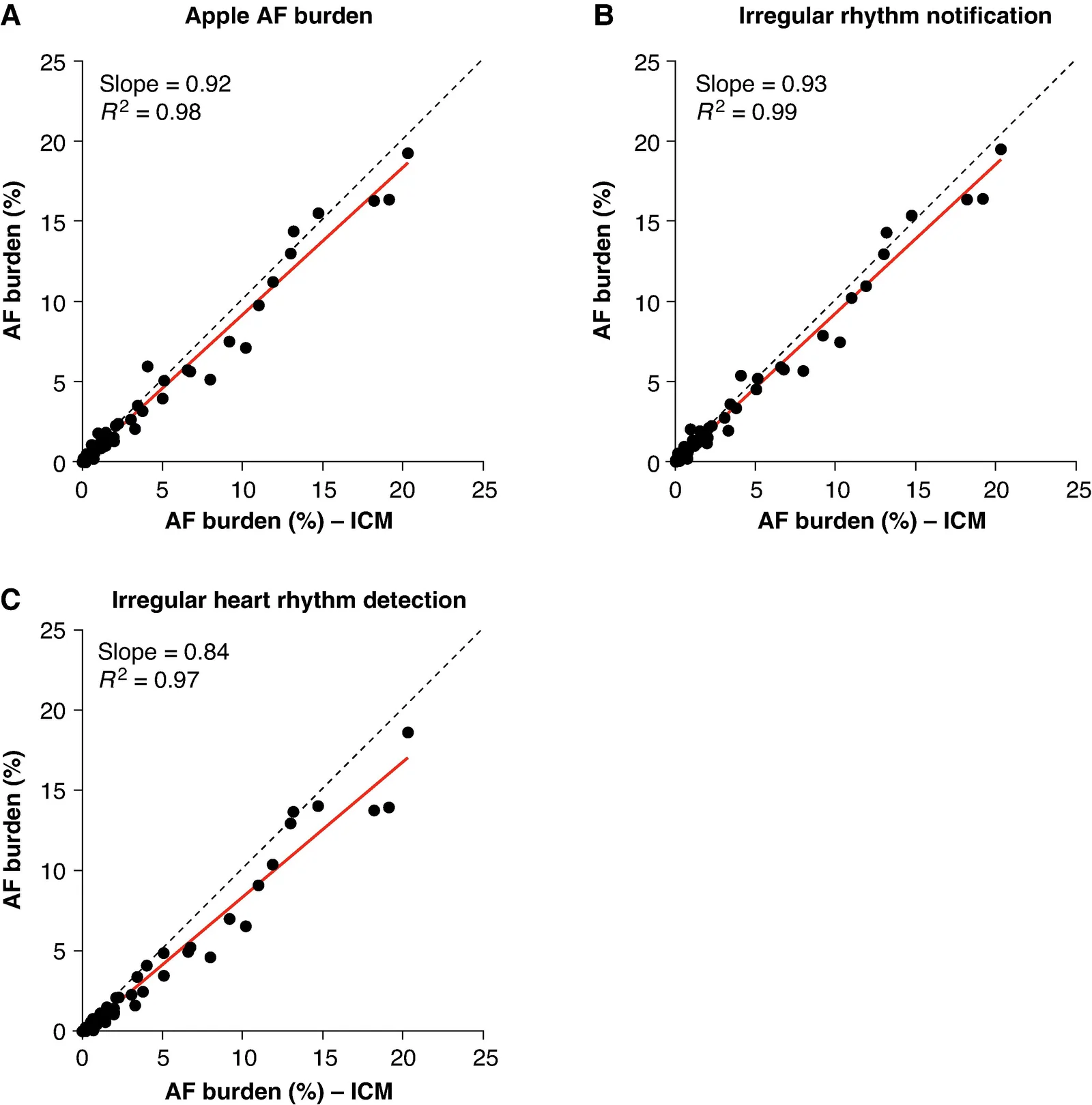
Correlation between wearable-estimated and ICM-derived AF burden. Scatter plots showing correlation between AF burden estimated by wearable algorithms and the reference ICM-derived burden. Pearson correlation coefficients (r) are displayed for each algorithm. All wearable algorithms demonstrate excellent correlation (r > 0.97) with the gold-standard ICM-derived AF burden. Dotted black lines represent lines of identity.

The correlation between the ICM-derived AF burden and the AF burden obtained with the Apple Watch AF burden algorithm, the Apple Watch IRN algorithm and the Fitbit IHRD algorithm was excellent for the study population as a whole (Pearson r, 0.98, 0.99 and 0.97, respectively; *Figure 3*) as well as when considering only patients with AT/AF recurrence (non-zero AF burden; Pearson r, 0.98, 0.99, 0.97, respectively; Supplementary material online, *Figure S1*). These results are summarized in *Table 1*.

**Table 1:** Summary of AF recurrence detection and burden estimation performance across monitoring strategies

	Arrhythmia-free survival (%)	Detection sensitivity (%)	Median AF burden (Recurrences only; %)	Correlation (Pearson r vs. ICM)	Median AF burden in missed detections (%)
ICM	52.6	100	0.15	—	—
Apple Watch AF Burden	61.3	82.2	0.13	0.98	<0.01
Apple Watch IRN	66.8	70.6	0.09	0.99	<0.01
Fitbit IHRD	69.7	64.4	0.05	0.97	<0.01
AECG 24 h (x3)	92.5	15.8	1.00	0.41	0.09
AECG 48 h (x3)	88.4	24.5	0.88	0.57	0.08
AECG 7d (x3)	79.8	41.6	0.36	0.75	0.06
AECG 14d (x3)	69.4	64.6	0.22	0.85	0.03

Comparison of arrhythmia-free survival, detection sensitivity, AF burden (in patients with recurrence), correlation with ICM-derived burden, and AF burden in missed detections across different monitoring modalities. Wearable smartwatch algorithms (Apple Watch AF Burden, Apple Watch IRN, Fitbit IHRD) were simulated using daytime use (8:00–22:00) and benchmarked against implantable cardiac monitors (ICMs) and commonly used intermittent ambulatory ECG (AECG) strategies. Values represent performance from day 90 to 365 post-ablation.

### Impact of daily watch usage on detection characteristics

**Figure 4:**
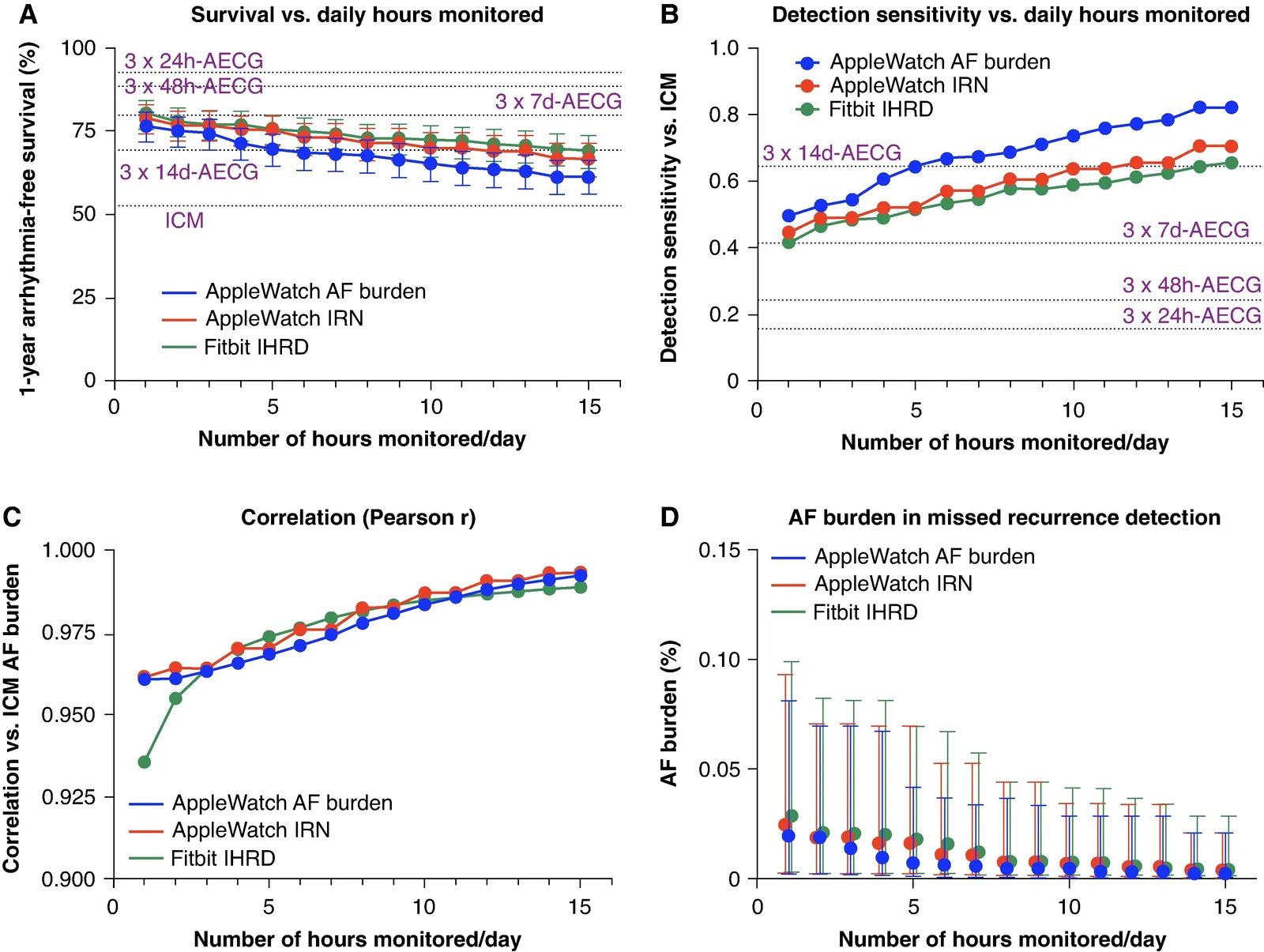
Effect of daily watch usage on detection and AF burden accuracy. (*A*) One-year arrhythmia-free survival as a function of daily watch usage (1–15 h/day) for each wearable algorithm. (*B*) Sensitivity for arrhythmia recurrence detection compared with ICM. (*C*) Correlation (Pearson r) between wearable- and ICM-derived AF burden across usage levels. (*D*) Median ICM-derived AF burden among patients with missed recurrence detection by daily watch usage. Longer daily wearable use substantially improves detection sensitivity without compromising accuracy of AF burden. Dotted lines indicate values for simulated non-invasive monitoring strategies.

Simulations of 1-year arrhythmia-free survival rates detected by wearable devices decreased with increasing daily watch usage from 76.6% (95% CI, 71.8–80.7%), 78.9% (95% CI, 74.2–82.8%) and 80.3% (95% CI, 75.8–84.2%) with 1 h of daily usage of Apple Watch AF burden algorithm, the Apple Watch IRN algorithm and the Fitbit IHRD algorithm, respectively, to 61.3% (55.9–66.2%), 66.8% (95% CI, 61.5–71.5%) and 69.1% (95% CI, 63.9–73.7%), respectively with 15 h of daily usage (*Figure 4A*). This translated to an increase in detection sensitivity (vs. ICM) from 0.50, 0.45 and 0.42 with the Apple Watch AF burden algorithm, the Apple Watch IRN algorithm and the Fitbit IHRD algorithm, respectively, with 1 h of daily watch usage to 0.82, 0.71 and 0.66, respectively, with 15 h of daily watch usage (*Figure 4B*). For the theoretical scenario of 24-hour of daily watch usage, the 1-year arrhythmia-free survival for the Apple Watch AF burden algorithm, the Apple Watch IRN algorithm and the Fitbit IHRD algorithm would have been of 57.5%. (95% CI, 52.5–62.9%), 63.0% (95% CI, 57.7–67.9%) and 63.9% (95% CI, 58.6–68.7%) (see Supplementary material online, *Figure S2*), respectively, returning sensitivities of 90.2%, 78,5% and 76.7%, respectively.

The correlation (Pearson r) between ICM-derived and watch-derived AF burden ranged from 0.96, 0.96 and 0.94 with 1 h of daily watch usage to 0.99, 0.99 and 0.99 with 15 h of daily watch usage for the Apple Watch AF burden algorithm, the Apple Watch IRN algorithm and the Fitbit IHRD algorithm, respectively (*Figure 4C*).

The ICM-derived AF burden in those patients with missed recurrence detection was very low, ranging from 0.020% (25–75th percentile, 0.003–0.082%), 0.025% (25–75th percentile, 0.003–0.093%) and 0.028% (25–75th percentile, 0.003–0.99%) with 1 h of daily watch usage to 0.003% (25–75th percentile, 0.001–0.021%), 0.004% (25–75th percentile, 0.001–0.021%) and 0.004 (25–75th percentile, 0.002–0.029%), with 15 h of daily watch usage with the Apple Watch AF burden algorithm, the Apple Watch IRN algorithm and the Fitbit IHRD algorithm, respectively (*Figure 4D*). The number of patients with missed recurrence detection and ICM-derived AF burden >0.1% ranged from 17/89 (19.1%), 21/89 (23.6%) and 24 (26.9%) with 1 h of daily watch usage to 0/89 (0%), 1/89 (1.1%) and 2/89 (2.2%), with 15 h of daily watch usage, with the Apple Watch AF burden algorithm, the Apple Watch IRN algorithm and the Fitbit IHRD algorithm, respectively.

### Impact of patient activity on detection characteristics

We simulated 4 different levels of patient activity corresponding to a 0% (‘None’), 25% (‘Low’), 50% (‘Intermediate’) and 75% (‘High’) probability that any given rhythm check would be missed because of patient activity.

**Figure 5:**
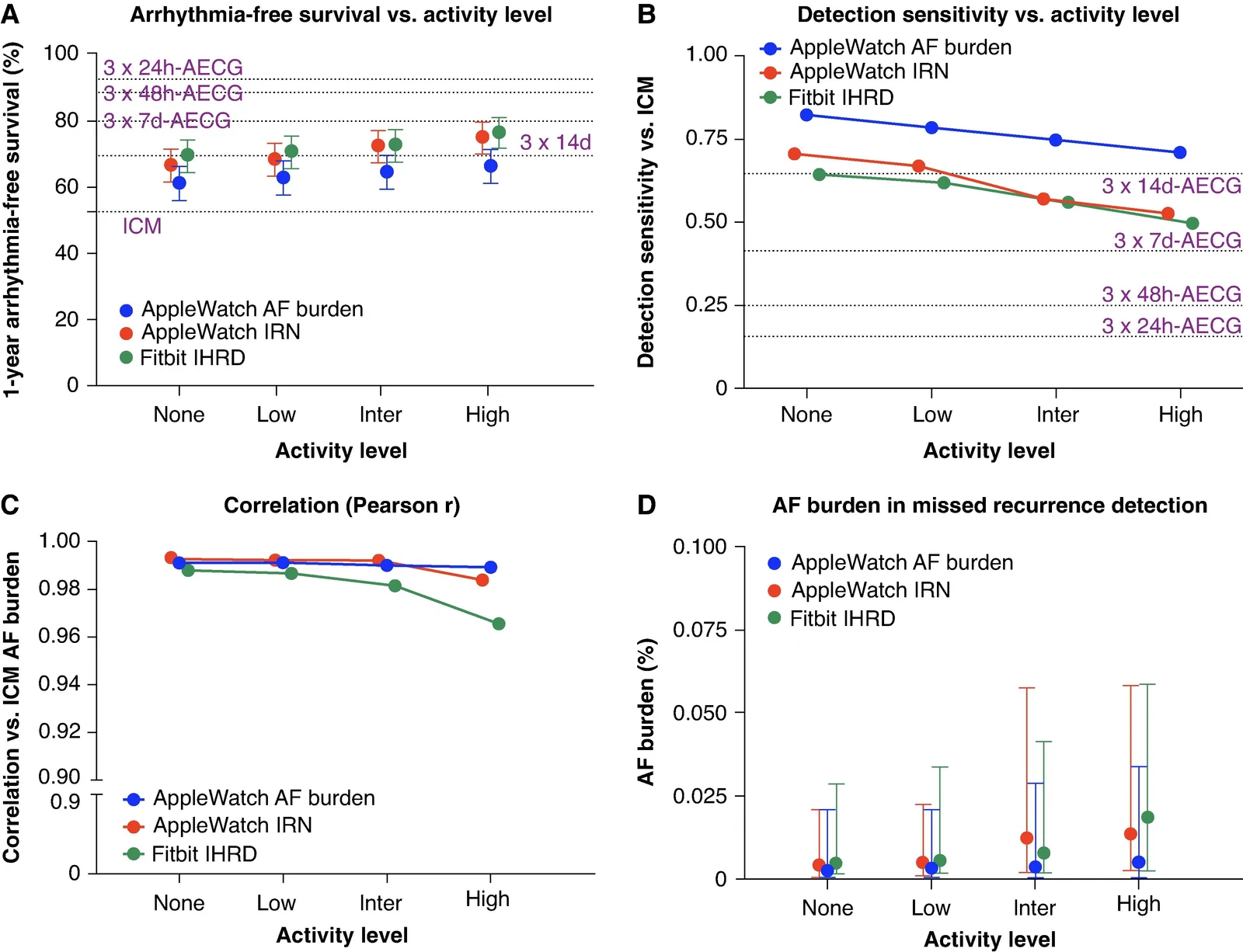
Impact of patient activity level on detection and burden estimation. (*A*) One-year arrhythmia-free survival by simulated patient activity level (0%, 25%, 50%, and 75% probability of missing checks). (*B*) Sensitivity for recurrence detection compared with ICM. (*C*) Correlation (Pearson r) between wearable- and ICM-derived AF burden across activity levels. (*D*) Median ICM-derived AF burden among patients with missed recurrence detection at each activity level. Detection performance remains robust despite high patient activity, with minimal clinically relevant missed episodes. Dotted lines indicate simulated non-invasive monitoring results.

The calculated 1-year arrhythmia-free survival increased with increasing activity levels (*Figure 5A*), ranging from 61.3% (95% CI, 55.9–66.2%), 66.8% (95% CI, 61.5–71.5%) and 69.7% (95% CI, 64.5–74.2%) with no patient activity (‘None’) to 66.5% (95% CI, 61.2–71.2%), 75.1% (95% CI, 70.2–79.4%) and 76.6% (95% CI, 71.8–80.7%) with the highest level of patient activity (‘High’), for the Apple Watch AF burden algorithm, the Apple Watch IRN algorithm and the Fitbit IHRD algorithm, respectively (*Figure 5A*). This corresponded to a decrease in detection sensitivity from 0.82, 0.71 and 0.64 with no patient activity (‘None’) to 0.71, 0.52 and 0.50 with the highest level of patient activity (‘High’), for the Apple Watch AF burden algorithm, the Apple Watch IRN algorithm and the Fitbit IHRD algorithm, respectively (*Figure 5B*).

The correlation (Pearson r) between the ICM-derived AF burden and the wearable-estimated AF burden remained >0.96 across all levels of patient activity, for all 3 algorithms (*Figure 5C*). The median ICM-derived AF burden in those patients with missed recurrence detection was <0.01% across all algorithms and activity levels (*Figure 5D*). The number of patients with missed recurrence detection and ICM-derived AF burden >0.1% was 0/89 (0%), 1/89 (1.1%) and 2/89 (2.2%) with no patient activity, 1/89 (1.1%), 2/89 (2.2%) and 3/89 (3.4%) with low patient activity and 2/89 (2.2%), 14/89 (15.7%) and 13/89 (14.6%), with the highest level of patient activity, for the Apple Watch AF burden algorithm, the Apple Watch IRN algorithm and the Fitbit IHRD algorithm, respectively. In additional simulations restricted to nocturnal wear (00:00–08:00), sensitivities were 72.4% for the Apple Watch AF burden algorithm, 62.6% for Apple Watch IRN, and 65.6% for Fitbit IHRD. These values were similar to those obtained during daytime use and in some scenarios outperformed daytime monitoring under high-noise/activity conditions.

## Discussion

The main findings of this study are that (i) wearable smartwatch algorithms specifically, the Apple Watch and Fitbit algorithms, showed strong sensitivity for detecting arrhythmia recurrence after AF ablation with detection sensitivities as high as 82%, when benchmarked against ICM; (ii) these algorithms delivered AF burden estimates which were strongly correlated with AF burden derived from continuous invasive monitoring (Pearson r > 0.97); and (iii) the AF burden in patients with missed detection by wearables was extremely low (median <0.01%). These findings support the integration of wearable devices as a viable, non-invasive strategy for rhythm monitoring following AF ablation.

### AF detection by wearables

An ever-growing number of direct-to-consumer wearable devices are available on the market. Several studies have evaluated the accuracy of smartwatch algorithms in detecting AF.^12–24^ The Apple Heart Study evaluated the AF detection capabilities of the Apple Watch in 419 297 participants without known AF—individuals who received an irregular heart rate notification were sent a 7-day ambulatory ECG patch.^25^ In this study population with a very low AF prevalence, the positive predictive value of an irregular heart rate notification was high (0.84; 95% CI, 0.76–0.92). Similarly, the Fitbit Heart Study employed a similar study design to evaluate Fitbit’s PPG-based algorithm, yielding favourable detection metrics (positive predictive value, 98.2%; sensitivity, 67.5%; specificity, 98.4%).^11^ Along the same line, the WATCH-AF trial reported a sensitivity of 93.7% and specificity of 98.2% for detecting AF with PPG-based wearables, using single-lead ECG as the comparator.^26^ A recent meta-analysis of 15 studies totalling 12 802 participants found a pooled sensitivity and specificity for AF detection for PPG-based smartwatch algorithms of 97.4% (95% CI, 96.5–98.3%) and 96.6% (95% CI, 94.9–98.3%), respectively.^27^

Importantly, all these studies have used intermittent non-invasive monitoring, in the form of Holter monitors or patches, as the benchmark against which wearables were compared. Hence, these reports should be interpreted within the significant limitations of non-invasive monitoring for detecting AF.^5^ How the AF detection capabilities of smartwatch algorithms compare to those of an ICM, the gold standard, is unknown. Furthermore, a large proportion of the aforementioned studies evaluated wearables for AF screening in individuals without known AF (characterized by a very low AF prevalence). We found a single study^28^ that assessed the AF recurrence detection capabilities of a smartwatch system (JKwear 1, Chengdu CVhealth Science and Technology Co., Ltd; not available in North America or Europe) specifically in the post-AF ablation setting, albeit using a 24-hour ECG patch monitor as the comparator.

To our knowledge, this is the first study to systematically evaluate the performance of wearable AF detection algorithms against ICMs in the post-AF ablation setting. Leveraging continuous, adjudicated rhythm data from the CIRCA-DOSE randomized trial, we simulated the performance of three commercially available smartwatch algorithms (Apple Watch AF Burden, Apple Watch IRN, and Fitbit IHRD) over a full year of follow-up. This design offers several key strengths. First, by benchmarking wearable detection against ICMs, the gold standard for arrhythmia surveillance, we provide the most rigorous validation of smartwatch-based monitoring to date. Second, we evaluated both recurrence detection and AF burden estimation, addressing the growing clinical emphasis on burden quantification as a therapeutic endpoint. Third, we examined the impact of real-world factors, such as variable daily watch usage and patient activity, on detection sensitivity and signal fidelity, thereby offering practical insights into algorithm robustness. Finally, by applying multiple algorithms to the same patient cohort, we were uniquely positioned to compare their operational characteristics under identical clinical conditions. Collectively, these strengths position our study as a critical step forward in validating wearable devices for post-ablation rhythm monitoring.

### Wearables vs. non-invasive intermittent monitoring

We found that wearables delivered good 1-year arrhythmia-free survival, recurrence detection sensitivities, and AF burden estimation. All three algorithms matched or outperformed three 14-day AECG monitors (worn at 3, 6 and 12 months), which is a much more intensive monitoring strategy than routine clinical practice or clinical trials. Moreover, even minimal watch usage (1 h per day) yielded an arrhythmia recurrence detection sensitivity superior to that of three 7-day AECG monitors. These findings are clinically relevant, given the widespread limitations of patch monitors, including poor patient compliance and frequent signal noise.

There is a growing interest in reporting AF burden in addition to the binary time-to-recurrence metric. We found that AF burden estimates derived from wearables are highly correlated (Pearson r > 0.97) with the gold-standard ICM-derived burden, even for high levels of patient activity or low daily watch usage. Furthermore, the AF burden in patients with missed recurrence detection was extremely low (median <0.02%), much below the 0.1% threshold for clinical relevance.^29^ Although we did not formally evaluate the cost-effectiveness of wearables for post-ablation arrhythmia detection, their superior arrhythmia detection characteristics and competitive pricing vs. non-invasive intermittent monitoring with Holter or ECG patches make wearables and *a priori* attractive option. Furthermore, smartwatches enable the tracking of other potentially clinically relevant metrics, such as pre- vs. post-ablation activity levels, which are not available with most Holter/patch systems.^30^

### AF detection algorithms

Our study was not designed to compare the algorithms with one another. All three algorithms displayed similar dependence on daily watch usage and patient activity level for optimal detection operational characteristics. The Apple Watch AF burden algorithm nevertheless had the highest arrhythmia detection across the different conditions evaluated in our study, whereas the Fitbit’s Irregular Heart Rate Detection algorithm had the lowest. This difference is likely the consequence of how the algorithms are designed. The Apple Watch algorithm checks for AF every 15 min and calculates the burden as the percentage of checks with an irregular rhythm. The Fitbit has a more stringent algorithm that requires 11 consecutive checks to have an irregular rhythm before generating a detection event. Given that a significant proportion of AF/AT episodes were of short duration (2 min), the Fitbit algorithm, which requires 11 consecutive 5-minute segments (staggered every 2.5 min), would miss arrhythmia episodes of duration less than 25 min. We were unable to directly evaluate how this more stringent criterion affects specificity. However, most reports evaluating the Apple Watch returned very high specificity values, suggesting that the less stringent Apple Watch algorithm retains good operational characteristics. Our data support the conclusion that frequent and systematic sampling—as modelled in the Apple Watch AF burden algorithm—yields more consistent estimates of AF burden than less frequent or alert-based strategies.

A limitation of PPG-based detection algorithms is non-interpretable tracings due to patient activity or inadequate watch placement. The proportion of non-interpretable tracings has been reported to be in the 20%-range.^31,32^ Zhao and colleagues reported a significant difference in the proportion of day vs. nighttime invalid tracings, with almost 60% of daytime tracings being reported as invalid for interpretation.^28^ We evaluated a wide range of simulated patient activity levels, corresponding to 0% to 75% of input tracings being considered invalid for interpretation. We found that the Apple Watch AF burden algorithm was fairly robust in terms of maintaining good AF/AT detection sensitivity and excellent AF burden correlation even with significantly degraded input data. Conversely, the Fitbit IHRD algorithm’s detection characteristics were impacted considerably by patient activity. Newer artificial intelligence-based smartwatch algorithms may enhance the diagnostic accuracy and utility of smartwatch ECGs for detecting atrial arrhythmias.^33,34^ Our findings align with the EHRA Practical Guide on the use of digital devices for arrhythmia management, which emphasizes the importance of tailoring monitoring strategies to patient characteristics, device capabilities, and real-world adherence considerations.^35^ Incorporating such guidance reinforces the potential role of smartwatch-based AF detection as part of a broader digital health ecosystem for post-ablation follow-up.

### Clinical implications

The findings of this study have important implications for post-ablation care in patients with AF. Smartwatch-based monitoring can significantly enhance post-ablation surveillance by improving detection of AF recurrence and accurately estimating AF burden compared with conventional strategies. Even modest daily use provided better recurrence detection than repeated Holter or patch monitors, which are resource-intensive and prone to patient non-compliance.

The extremely low AF burden in missed cases (<0.02%) suggests that undetected episodes are unlikely to be clinically meaningful. These results support the integration of wearables into clinical care pathways as a cost-effective, patient-centred alternative to intermittent monitoring, with potential to optimize follow-up, reduce unnecessary testing, and inform guideline recommendations for post-ablation monitoring.

In contrast to 14-day patch monitors or event recorders, which offer only intermittent snapshots, wearables provide an opportunity for continuous, scalable, and patient-directed rhythm surveillance. Furthermore, the deployment of smartwatches enables a wide range of digital AF surveillance paradigms, including tracking physical activity, sleep, and heart rate variability, which provides a multidimensional perspective on recovery following ablation. These functions can facilitate the early identification of AF recurrence, promote lifestyle interventions, and enable remote clinical decision-making to enhance patient outcomes and optimize healthcare resource utilization.

The clinical importance of AF burden assessment has been underscored in the recent ESC/EHRA consensus statement, which defines AF burden as the proportion of time spent in AF during a specified monitoring period.^36^ AF burden has been linked to stroke risk, heart failure progression, cognitive decline, renal dysfunction, and mortality, and is increasingly recognized as a meaningful endpoint in clinical practice and research.^29,37–42^ Recent ablation trials have begun to incorporate AF burden reduction, rather than recurrence alone, as an outcome, reflecting that substantial reductions—even without complete elimination—can yield meaningful clinical benefit. Our findings, showing excellent correlation between smartwatch-estimated and ICM-derived AF burden, suggest that consumer wearables may offer a scalable, non-invasive method for capturing this clinically relevant endpoint in both trials and routine follow-up.

## Limitations

This study has several limitations. First, the simulations were based on retrospective application of wearable detection algorithms to ICM-derived rhythm data. As such, this study represents an ‘ideal state’ comparison of the wearables, assuming ideal compliance with wearable use (i.e. worn daily) and performance of the PPG-based detection algorithms. A significant limitation of wearable devices is limited compliance (e.g. < 40% of those with cardiovascular disease report daily use), the relatively high rate of inconclusive diagnoses (up to 25% of episodes), as well as false-positive and false-negative detections of AF. The impact of over- and under-detection, especially in noisy signal conditions or during exercise, remains to be investigated. However, a recent real-world validation study in patients with established AF demonstrated that PPG-based monitoring was generally reliable, with the main limitation being a modest overestimation of heart rate.^43^ Second, we assumed wearing during daytime from 8:00 a.m. to 10:00 p.m., excluding overnight monitoring. This creates a bias against wearable-based detection, but it is also the most common usage of smartwatches, given that they must be charged. Third, the studied algorithms were based on public documentation descriptions. Although these simulations model their functioning, we cannot account for proprietary software improvements or future algorithmic developments. Fourth, for modelling purposes, all atrial tachyarrhythmias (AF, atrial flutter, and atrial tachycardia) were analysed collectively; however, the performance of the smartwatch algorithm may vary between AF and organized atrial tachyarrhythmias. However, only 3 of the 163 (1.8%) patients with arrhythmia recurrence had recurrence only in the form of AT/AFL. Moreover, the rate of recurrence in the form of AT/AFL is less after ablation of paroxysmal vs. persistent AF. Fifth, the patient population in this study had paroxysmal AF, and it remains unknown whether our findings can be extrapolated to those with persistent AF or those with different degrees of AF burden. Finally, estimating AF burden with smartwatches may require a certain degree of digital literacy, which could limit widespread implementation in real-world practice, particularly among older patients.^44^

## Conclusion

Wearable ECG-based smartwatches demonstrate an excellent correlation with implantable cardiac monitors for assessing AF burden and present reasonable sensitivity for detecting post-ablation arrhythmia recurrences. These findings support the integration of wearable ECG devices into post-ablation follow-up strategies, offering a scalable alternative to traditional monitoring.

## Supplementary Material

euaf280_Supplementary_Data

## References

[1] Linz D, Gawalko M, Betz K, Hendriks JM, Lip GYH, Vinter N et al Atrial fibrillation: epidemiology, screening and digital health. Lancet Reg Health Eur 2024;37:100786. 38362546 10.1016/j.lanepe.2023.100786 PMC10866942

[2] Tzeis S, Gerstenfeld EP, Kalman J, Saad EB, Sepehri Shamloo A, Andrade JG et al 2024 European Heart Rhythm Association/Heart Rhythm Society/Asia Pacific Heart Rhythm Society/Latin American Heart Rhythm Society expert consensus statement on catheter and surgical ablation of atrial fibrillation. Europace 2024;26:euae043. 38587017 10.1093/europace/euae043 PMC11000153

[3] Aguilar M, Macle L, Ditac G, Benali K, Honfo SH, Khairy P et al Atrial fibrillation burden is underestimated by non-invasive monitoring: the CIRCA-DOSE trial. Eur Heart J 2025:ehaf647. 10.1093/eurheartj/ehaf647 40972515

[4] Kalarus Z, Mairesse GH, Sokal A, Boriani G, Sredniawa B, Casado-Arroyo R et al Searching for atrial fibrillation: looking harder, looking longer, and in increasingly sophisticated ways. An EHRA position paper. Europace 2023;25:185–98. 36256580 10.1093/europace/euac144 PMC10112840

[5] Aguilar M, Macle L, Deyell MW, Yao R, Hawkins NM, Khairy P et al Influence of monitoring strategy on assessment of ablation success and postablation atrial fibrillation burden assessment: implications for practice and clinical trial design. Circulation 2022;145:21–30. 34816727 10.1161/CIRCULATIONAHA.121.056109

[6] Francisco A, Pascoal C, Lamborne P, Morais H, Goncalves M. Wearables and atrial fibrillation: advances in detection, clinical impact, ethical concerns, and future perspectives. Cureus 2025;17:e77404. 39949464 10.7759/cureus.77404 PMC11822239

[7] Dhingra LS, Aminorroaya A, Oikonomou EK, Nargesi AA, Wilson FP, Krumholz HM et al Use of wearable devices in individuals with or at risk for cardiovascular disease in the US, 2019 to 2020. JAMA Netw Open 2023;6:e2316634. 37285157 10.1001/jamanetworkopen.2023.16634 PMC10248745

[8] Andrade JG, Champagne J, Dubuc M, Deyell MW, Verma A, Macle L et al Cryoballoon or radiofrequency ablation for atrial fibrillation assessed by continuous monitoring: a randomized clinical trial. Circulation 2019;140:1779–88. 31630538 10.1161/CIRCULATIONAHA.119.042622

[9] Andrade JG, Deyell MW, Badra M, Champagne J, Dubuc M, Leong-Sit P et al Randomised clinical trial of cryoballoon versus irrigated radio frequency catheter ablation for atrial fibrillation-the effect of double short versus standard exposure cryoablation duration during pulmonary vein isolation (CIRCA-DOSE): methods and rationale. BMJ Open 2017;7:e017970. 10.1136/bmjopen-2017-017970 PMC5639989 28982836

[10] Inc A. Using Apple Watch for Arrhythmia Detection. https://www.apple.com/healthcare/docs/site/Apple_Watch_Arrhythmia_Detection.pdf 2025 date last accessed).

[11] Lubitz SA, Faranesh AZ, Selvaggi C, Atlas SJ, McManus DD, Singer DE et al Detection of atrial fibrillation in a large population using wearable devices: the Fitbit Heart Study. Circulation 2022;146:1415–24. 36148649 10.1161/CIRCULATIONAHA.122.060291 PMC9640290

[12] Santala OE, Lipponen JA, Jantti H, Rissanen TT, Tarvainen MP, Laitinen TP et al Continuous mHealth patch monitoring for the algorithm-based detection of atrial fibrillation: feasibility and diagnostic accuracy study. JMIR Cardio 2022;6:e31230. 35727618 10.2196/31230 PMC9257607

[13] Selder JL, Te Kolste HJ, Twisk J, Schijven M, Gielen W, Allaart CP. Accuracy of a standalone atrial fibrillation detection algorithm added to a popular wristband and smartwatch: prospective diagnostic accuracy study. J Med Internet Res 2023;25:e44642. 37234033 10.2196/44642 PMC10257103

[14] Nonoguchi NM, Soejima K, Goda A, Nishimura K, Onozuka D, Fujita S et al Accuracy of wristwatch-type photoplethysmography in detecting atrial fibrillation in daily life(). Eur Heart J Digit Health 2022;3:455–64. 36712156 10.1093/ehjdh/ztac041 PMC9707983

[15] Bashar SK, Han D, Ding E, Whitcomb C, McManus DD, Chon KH. Smartwatch based atrial fibrillation detection from photoplethysmography signals. Annu Int Conf IEEE Eng Med Biol Soc 2019;2019:4306–9. 31946820 10.1109/EMBC.2019.8856928

[16] Bashar SK, Han D, Hajeb-Mohammadalipour S, Ding E, Whitcomb C, McManus DD et al Atrial fibrillation detection from wrist photoplethysmography signals using smartwatches. Sci Rep 2019;9:15054. 31636284 10.1038/s41598-019-49092-2 PMC6803677

[17] Bonomi AG, Schipper F, Eerikainen LM, Margarito J, van Dinther R, Muesch G et al Atrial fibrillation detection using a novel cardiac ambulatory monitor based on photo-plethysmography at the wrist. J Am Heart Assoc 2018;7:e009351. 30371247 10.1161/JAHA.118.009351 PMC6201454

[18] Liao MT, Yu CC, Lin LY, Pan KH, Tsai TH, Wu YC et al Impact of recording length and other arrhythmias on atrial fibrillation detection from wrist photoplethysmogram using smartwatches. Sci Rep 2022;12:5364. 35354873 10.1038/s41598-022-09181-1 PMC8967835

[19] Han D, Bashar SK, Mohagheghian F, Ding E, Whitcomb C, McManus DD et al Premature atrial and ventricular contraction detection using photoplethysmographic data from a smartwatch. Sensors (Basel) 2020;20:5683. 33028000 10.3390/s20195683 PMC7582300

[20] Tison GH, Sanchez JM, Ballinger B, Singh A, Olgin JE, Pletcher MJ et al Passive detection of atrial fibrillation using a commercially available smartwatch. JAMA Cardiol 2018;3:409–16. 29562087 10.1001/jamacardio.2018.0136 PMC5875390

[21] Ding EY, Han D, Whitcomb C, Bashar SK, Adaramola O, Soni A et al Accuracy and usability of a novel algorithm for detection of irregular pulse using a smartwatch among older adults: observational study. JMIR Cardio 2019;3:e13850. 31758787 10.2196/13850 PMC6834225

[22] Lai D, Bu Y, Su Y, Zhang X, Ma CS. Non-standardized patch-based ECG lead together with deep learning based algorithm for automatic screening of atrial fibrillation. IEEE J Biomed Health Inform 2020;24:1569–78. 32175879 10.1109/JBHI.2020.2980454

[23] Shao M, Zhou Z, Bin G, Bai Y, Wu S. A wearable electrocardiogram telemonitoring system for atrial fibrillation detection. Sensors (Basel) 2020;20:606. 31979184 10.3390/s20030606 PMC7038204

[24] Chang PC, Wen MS, Chou CC, Wang CC, Hung KC. Atrial fibrillation detection using ambulatory smartwatch photoplethysmography and validation with simultaneous Holter recording. Am Heart J 2022;247:55–62. 35131229 10.1016/j.ahj.2022.02.002

[25] Perez MV, Mahaffey KW, Hedlin H, Rumsfeld JS, Garcia A, Ferris T et al Large-scale assessment of a smartwatch to identify atrial fibrillation. N Engl J Med 2019;381:1909–17. 31722151 10.1056/NEJMoa1901183 PMC8112605

[26] Dorr M, Nohturfft V, Brasier N, Bosshard E, Djurdjevic A, Gross S et al The WATCH AF trial: smartWATCHes for detection of atrial fibrillation. JACC Clin Electrophysiol 2019;5:199–208. 30784691 10.1016/j.jacep.2018.10.006

[27] Sibomana O, Hakayuwa CM, Obianke A, Gahire H, Munyantore J, Chilala MM. Diagnostic accuracy of ECG smart chest patches versus PPG smartwatches for atrial fibrillation detection: a systematic review and meta-analysis. BMC Cardiovasc Disord 2025;25:132. 40000931 10.1186/s12872-025-04582-2 PMC11853970

[28] Zhao Z, Li Q, Li S, Guo Q, Bo X, Kong X et al Evaluation of an algorithm-guided photoplethysmography for atrial fibrillation burden using a smartwatch. Pacing Clin Electrophysiol 2024;47:511–7. 38407298 10.1111/pace.14951

[29] Andrade JG, Deyell MW, Macle L, Steinberg JS, Glotzer TV, Hawkins NM et al Healthcare utilization and quality of life for atrial fibrillation burden: the CIRCA-DOSE study. Eur Heart J 2023;44:765–76. 36459112 10.1093/eurheartj/ehac692

[30] Nonoguchi NM, Soejima K, Katsume Y, Hoshida K, Togashi I, Goda A et al Wristwatch pulse wave monitoring: assessing daily activity post-catheter ablation for atrial fibrillation. Eur Heart J Digit Health 2025;6:96–103. 39846064 10.1093/ehjdh/ztae091 PMC11750189

[31] Mannhart D, Lischer M, Knecht S, du Fay de Lavallaz J, Strebel I, Serban T et al Clinical validation of 5 direct-to-consumer wearable smart devices to detect atrial fibrillation: BASEL wearable study. JACC Clin Electrophysiol 2023;9:232–42. 36858690 10.1016/j.jacep.2022.09.011

[32] Abu-Alrub S, Strik M, Ramirez FD, Moussaoui N, Racine HP, Marchand H et al Smartwatch electrocardiograms for automated and manual diagnosis of atrial fibrillation: a comparative analysis of three models. Front Cardiovasc Med 2022;9:836375. 35187135 10.3389/fcvm.2022.836375 PMC8854369

[33] Avram R, Ramsis M, Cristal AD, Nathan V, Zhu L, Kim J et al Validation of an algorithm for continuous monitoring of atrial fibrillation using a consumer smartwatch. Heart Rhythm 2021;18:1482–90. 33838317 10.1016/j.hrthm.2021.03.044

[34] Fiorina L, Chemaly P, Cellier J, Said MA, Coquard C, Younsi S et al Artificial intelligence-based electrocardiogram analysis improves atrial arrhythmia detection from a smartwatch electrocardiogram. Eur Heart J Digit Health 2024;5:535–41. 39318690 10.1093/ehjdh/ztae047 PMC11417483

[35] Svennberg E, Tjong F, Goette A, Akoum N, Di Biase L, Bordachar P et al How to use digital devices to detect and manage arrhythmias: an EHRA practical guide. Europace 2022;24:979–1005. 35368065 10.1093/europace/euac038 PMC11636571

[36] Doehner W, Boriani G, Potpara T, Blomstrom-Lundqvist C, Passman R, Sposato LA et al Atrial fibrillation burden in clinical practice, research, and technology development: a clinical consensus statement of the European Society of Cardiology council on stroke and the European heart rhythm association. Europace 2025;27:euaf019. 40073206 10.1093/europace/euaf019 PMC11901050

[37] Schwennesen HT, Andrade JG, Wood KA, Piccini JP. Ablation to reduce atrial fibrillation burden and improve outcomes: JACC review topic of the week. J Am Coll Cardiol 2023;82:1039–50. 37648353 10.1016/j.jacc.2023.06.029 PMC11103629

[38] Turakhia MP, Ziegler PD, Schmitt SK, Chang Y, Fan J, Than CT et al Atrial fibrillation burden and short-term risk of stroke: case-crossover analysis of continuously recorded heart rhythm from cardiac electronic implanted devices. Circ Arrhythm Electrophysiol 2015;8:1040–7. 26175528 10.1161/CIRCEP.114.003057

[39] Piccini JP, Passman R, Turakhia M, Connolly AT, Nabutovsky Y, Varma N. Atrial fibrillation burden, progression, and the risk of death: a case-crossover analysis in patients with cardiac implantable electronic devices. Europace 2019;21:404–13. 30462208 10.1093/europace/euy222

[40] Samuel M, Khairy P, Champagne J, Deyell MW, Macle L, Leong-Sit P et al Association of atrial fibrillation burden with health-related quality of life after atrial fibrillation ablation: substudy of the cryoballoon vs contact-force atrial fibrillation ablation (CIRCA-DOSE) randomized clinical trial. JAMA Cardiol 2021;6:1324–8. 34406350 10.1001/jamacardio.2021.3063 PMC8374730

[41] Steinberg BA, Li Z, O'Brien EC, Pritchard J, Chew DS, Bunch TJ et al Atrial fibrillation burden and heart failure: data from 39,710 individuals with cardiac implanted electronic devices. Heart Rhythm 2021;18:709–16. 33508517 10.1016/j.hrthm.2021.01.021 PMC8096675

[42] Reddy VY, Gerstenfeld EP, Schmidt B, Andrade JG, Nair D, Natale A et al Pulsed field ablation of persistent atrial fibrillation with continuous ECG monitoring follow-up: ADVANTAGE AF-phase 2. Circulation 2025;152:27–40. 40273320 10.1161/CIRCULATIONAHA.125.074485 PMC12225731

[43] Gruwez H, Ezzat D, Van Puyvelde T, Dhont S, Meekers E, Bruckers L et al Real-world validation of smartphone-based photoplethysmography for rate and rhythm monitoring in atrial fibrillation. Europace 2024;26:euae065. 38630867 10.1093/europace/euae065 PMC11023210

[44] Svennberg E, Caiani EG, Bruining N, Desteghe L, Han JK, Narayan SM et al The digital journey: 25 years of digital development in electrophysiology from an Europace perspective. Europace 2023;25:euad176. 37622574 10.1093/europace/euad176 PMC10450797

