# Supplementary material for: Wearable smartwatches for atrial fibrillation detection and burden estimation after ablation: comparison with continuous monitoring
Source: Europace. 2025 Nov 10;28(9):euaf280. doi: 10.1093/europace/euaf280 (PMC13564214; doi:10.1093/europace/euaf280)
Supplement: Supplementary file 1 [file euaf280_supplementary_data.docx]

**SUPPLEMENTAL MATERIAL**

**Wearable Smartwatches for Atrial Fibrillation Detection and Burden Estimation After Ablation: Comparison With Continuous Monitoring**

Martin Aguilar MD PhD^1^, Laurent Macle MD^1^, Ralph Chamieh MD^1^, Paul Khairy MD PhD^1^, Marc W. Deyell MD MSc^2,3^, Richard G. Bennett BSc, MBChB, PhD^2,3^, Jason G. Andrade MD^1,2,3^

**Supplemental Figure 1.** Correlation among patients with non-zero AF burden. Correlation between wearable-estimated and ICM-derived AF burden restricted to patients with documented recurrence. Pearson r values shown for each algorithm.

**Supplemental Figure 2.** Simulated 24-hour wearable usage. One-year arrhythmia-free survival if wearables were worn continuously (24 hours/day), compared with ICM and across all three wearable algorithms.
